# Clinical-Pharmacogenetic Predictive Models for Time to Occurrence of Levodopa Related Motor Complications in Parkinson’s Disease

**DOI:** 10.3389/fgene.2019.00461

**Published:** 2019-05-16

**Authors:** Sara Redenšek, Barbara Jenko Bizjan, Maja Trošt, Vita Dolžan

**Affiliations:** ^1^Pharmacogenetics Laboratory, Institute of Biochemistry, Faculty of Medicine, University of Ljubljana, Ljubljana, Slovenia; ^2^Department of Neurology, University Medical Centre Ljubljana, Ljubljana, Slovenia

**Keywords:** Parkinson’s disease, polymorphisms, pharmacogenetics, personalized medicine, motor fluctuations, dyskinesia, dopaminergic treatment

## Abstract

The response to dopaminergic treatment in Parkinson’s disease depends on many clinical and genetic factors. The very common motor fluctuations (MF) and dyskinesia affect approximately half of patients after 5 years of treatment with levodopa. We did an evaluation of a combined effect of 16 clinical parameters and 34 single nucleotide polymorphisms to build clinical and clinical-pharmacogenetic models for prediction of time to occurrence of motor complications and to compare their predictive abilities. In total, 220 Parkinson’s disease patients were included in the analysis. Their demographic, clinical, and genotype data were obtained. The combined effect of clinical and genetic factors was assessed using The Least Absolute Shrinkage and Selection Operator penalized regression in the Cox proportional hazards model. Clinical and clinical-pharmacogenetic models were constructed. The predictive capacity of the models was evaluated with the cross-validated area under time-dependent receiver operating characteristic curve. Clinical-pharmacogenetic model included age at diagnosis (HR = 0.99), time from diagnosis to initiation of levodopa treatment (HR = 1.24), *COMT* rs165815 (HR = 0.90), *DRD3* rs6280 (HR = 1.03), and *BIRC5* rs9904341 (HR = 0.95) as predictive factors for time to occurrence of MF. Furthermore, clinical-pharmacogenetic model for prediction of time to occurrence of dyskinesia included female sex (HR = 1.07), age at diagnosis (HR = 0.97), tremor-predominant Parkinson’s disease (HR = 0.88), beta-blockers (HR = 0.95), alcohol consumption (HR = 0.99), time from diagnosis to initiation of levodopa treatment (HR = 1.15), *CAT* rs1001179 (HR = 1.27), *SOD2* rs4880 (HR = 0.95), *NOS1* rs2293054 (HR = 0.99), *COMT* rs165815 (HR = 0.92), and *SLC22A1* rs628031 (HR = 0.80). Areas under the curves for clinical and clinical-pharmacogenetic models for MF after 5 years of levodopa treatment were 0.68 and 0.70, respectively. Areas under the curves for clinical and clinical-pharmacogenetic models for dyskinesia after 5 years of levodopa treatment were 0.71 and 0.68, respectively. These results show that clinical-pharmacogenetic models do not have better ability to predict time to occurrence of motor complications in comparison to the clinical ones despite the significance of several polymorphisms. Models could be improved by a larger sample size and by additional polymorphisms, epigenetic predictors or serum biomarkers.

## Introduction

The loss of dopaminergic neurons in the nigrostriatal pathway is one of the main pathological hallmarks of Parkinson’s disease (PD). Consequential dopamine depletion calls for dopamine replacement, which is the main strategy of symptomatic treatment of PD. The gold standard of PD management is levodopa. However, long-term treatment may lead to motor complications, such as MF and dyskinesia. MF present as oscillations between good motor symptom control and reduced motor symptom control, while dyskinesia manifest as involuntary choreatic or dystonic movements (Kalia and Lang, 2015). In general, motor complications develop in 30% of patients after 2–3 years of exposure to levodopa and in more than 50% after 5 years of exposure (Poewe et al., 2017). Genetic variability between patients may cause differences in the disease pathogenesis or in the pharmacokinetics and pharmacodynamics of levodopa. Along with different clinical parameters, this variability may determine the time to motor complications development after initiation of levodopa treatment.

The dopaminergic pathway is the most important effector pathway of PD pathogenesis. Genetic defects in different parts of the pathway, such as metabolic enzymes (*COMT, DDC, MAOB*), transporters (*SLC6A3, SLC22A1, SLC18A2, SLC7A5*), receptors (*DRD1-5*), and vesicle formation players (*SV2C*), may affect the disease occurrence, progression, and treatment response (Politi et al., 2018; Redenšek et al., 2019).

It is generally accepted that defects in neuroinflammatory and oxidative stress related pathways contribute to PD pathogenic processes (Poewe et al., 2017). Chronically activated microglia in the PD brain releases pro-inflammatory cytokines (IL1B, TNF, IL6) and reactive oxygen species among other entities. Several SNP in the *IL1B* (Lee et al., 2016)*, TNF* (Dai et al., 2014), and *IL6* (San Luciano et al., 2012) have already been associated with PD susceptibility. The activity of inflammasome NLRP3, which is responsible for IL1B production, has also been associated with PD (Sarkar et al., 2017). Furthermore, several anti-oxidant and pro-oxidant enzymes have been associated with PD susceptibility in terms of genetic variability and activity, such as glutathione peroxidase (*GPX1*) (Power and Blumbergs, 2009), catalase (*CAT*) (de Farias et al., 2016), superoxide dismutase (*SOD2*) (Santiago et al., 2014), and nitric oxide synthase (*NOS1*) (Rife et al., 2009).

Neurodevelopmental genes may also play a role in the PD development and progression. Neuregulin-1 (*NRG1*) has been associated with neurodegeneration and has been proposed as protective against neuroinflammation and oxidative stress (Xu et al., 2017). Genetic variability of *BDNF*, *NOTCH4*, and *BIRC5* may also influence PD by affecting developmental pathways of neurons, including their proliferation, differentiation, and apoptosis (Baratchi et al., 2010; Terzic et al., 2015). *BDNF* rs6265 has already been associated with the occurrence of dyskinesia (Foltynie et al., 2009).

A growing number of studies report different clinical and genetic factors influencing the occurrence and time to occurrence of motor complications (Sharma et al., 2010; Agundez et al., 2013; Eusebi et al., 2018; Redenšek et al., 2018). However, there is lack of studies evaluating joint contribution of different types of factors to development of a certain phenotype. Furthermore, discrepancies in the results hinder their translation to clinical practice. Therefore, a great need exists for a more comprehensive evaluation of clinical and genetic factors influencing the occurrence of motor complications. When limited amount of participants experience the adverse event (AE), special modeling approaches have to be adopted to enable inclusion of several explanatory variables in the statistical models. The LASSO penalized regression is one of the possible approaches. It presents a strict variable selection method to obtain results that are easy to interpret and are clinically more useful. It also minimizes the problem of overfitting, which makes results more reproducible and realistic (Moons et al., 2004; Goeman, 2010; Jenko et al., 2017).

The aim of this study was to evaluate the joint effect of demographic, clinical, and genetic factors on the time to occurrence of MF and dyskinesia after initiation of levodopa treatment. We wanted to build predictive models with as good as possible predictive capacity. In the final evaluation we tested the clinical parameters and polymorphisms from pathways of (i) dopamine metabolism, transport, and signaling, (ii) neuroinflammation, (iii) oxidative stress, (iv) neuron development, proliferation, and differentiation, and (v) apoptosis. We used LASSO penalized regression method to obtain predictive statistical models. Predictive capacity of the models was evaluated using the area under the time-dependent ROC curve (tAUC), which was properly cross-validated.

## Materials and Methods

### Participants and Clinical Data

Patients that fulfilled the following criteria were included in the study: (i) diagnosis of PD according to the UK Parkinson Disease Society Brain Bank criteria by an experienced movement disorders specialist (Goetz et al., 2008); (ii) available clinical data; (iii) at least 3 months of levodopa treatment duration; (iv) ongoing dopaminergic therapy. The recruitment period lasted from October 2016 to April 2018. There were 231 PD patients included in the study. Demographic and clinical data were collected with structured interviews with patients and their caregivers and from medical records.

The following demographic and clinical data were collected: (i) demographics (sex, age at diagnosis), (ii) disease specifics (tremor-predominant PD or other; body side of disease initiation), (iii) prodromal signs (REM sleep behaviour disorder, depression, constipation, olfactory dysfunction), (iv) treatment onset time (time from diagnosis to initiation of levodopa treatment), (v) co-medication (beta-blockers, non-steroidal anti-inflammatory drugs, calcium channel blockers, and statins), (vi) lifestyle data (tobacco smoking, alcohol consumption, and coffee consumption). Only explanatory variables that are known at the first levodopa prescription were included in the analysis to enable algorithm construction for potential therapy guiding.

The primary endpoints of the study were motor complications due to levodopa treatment, i.e., MF and dyskinesia. The time to occurrence of motor complications after initiation of levodopa was recorded.

The study protocol was approved by the Slovenian Ethics Committee for Research in Medicine (KME 42/05/16). All subjects gave written informed consent in accordance with the Declaration of Helsinki.

### SNP Selection

We thoroughly searched the literature for SNPs in genes that were shown to be involved in the dopamine metabolism, transport, and signaling, neuroinflammation, oxidative stress, neuron development, proliferation, differentiation, and apoptosis. We chose 37 SNPs in 23 candidate genes with a previously reported association with processes of neurodegeneration, PD pathogenesis, and dopaminergic treatment response (Baratchi et al., 2010; Terzic et al., 2015; Xu et al., 2017; Redenšek et al., 2018, 2019). Only functional polymorphisms with experimentally observed or *in silico* predicted function were included (Xu and Taylor, 2009). We included SNPs with minor allele frequency of at least 2%.

### Genotyping Analysis

Peripheral blood samples were obtained for DNA extraction. Genomic DNA was isolated using the FlexiGene DNA Kit (Qiagen, Hilden, Germany) according to the manufacturer’s protocol. All patients were genotyped for 37 SNPs in the genes involved in the processes of neuroinflammation: *NLRP3* (rs35829419), *CARD8* (rs2043211), *IL1B* (rs16944, rs1143623), *TNF* (rs1800629), and *IL6* (rs1800795); oxidative stress: *GPX1* (rs1050450), *CAT* (rs10836235, rs1001179), *SOD2* (rs4880), and *NOS1* (rs2293054, rs2682826); dopaminergic pathway: *COMT* (rs4680, rs165815), *DDC* (rs921451, rs3837091), *MAOB* (rs1799836), *SLC6A3* (rs393795, rs6347, rs104209), *SLC22A1* (rs628031), *SLC18A2* (rs14240), *SLC7A5* (rs1060253 and rs1060257), *DRD2* (rs1799732, rs1801028), *DRD3* (rs6280), *SV2C* (rs1423099); neuron development, proliferation, and differentiation: *BDNF* (rs6265), *NOTCH4* (rs367398), *NRG1* (rs10503929, rs3735782, rs3735781, rs3924999); and apoptosis: *BIRC5* (rs9904341, rs8073069, rs1787467). Thirty-four of the SNPs were genotyped with KASPar assays (KBiosciences, Herts, United Kingdom and LGC Genomics, United Kingdom) according to manufacturer’s instructions. Three of the SNPs (*TNF* rs1800629, *GPX1* rs1050450, and *CAT* rs10836235) were genotyped with TaqMan genotyping assays (Applied Biosystems, Foster City, CA, United States) also according to the manufacturer’s protocol. 10% of samples were genotyped in duplicate as quality control and all the results were concordant.

### Statistical Analysis

Median and 25th to 75th percentile range were used to describe central tendency and variability of continuous variables. Frequency and percentage were used to describe categorical variables. The agreement of genotype frequencies with HWE was evaluated by chi-squared test. The additive genetic model was used in all analyses.

Cox proportional hazards models were used to estimate the association of genetic polymorphisms and clinical covariates with the time to occurrence of motor complications after levodopa treatment initiation. Results were reported as *p*-values, HR and 95% CI. Bonferroni correction was used to account for multiple comparisons to prevent false positive results. The significance threshold was set to 0.001 (0.05/50). *P*-values up to 0.001 were considered statistically significant, while *p* values between 0.001 and 0.050 were considered nominally significant.

Clinical models including only clinical variables were built using Cox analysis with LASSO penalization. Clinical-pharmacogenetic models including clinical and genetic variables were built in similar way. Method of LASSO penalization was used due to a large amount of explanatory variables in comparison to the number of events. LASSO penalization also prevents overfitting to avoid over-optimistic results and shrinks the estimates of the regression coefficients obtained by the Cox regression toward zero. The shrinkage is estimated by the tuning parameter λ, which is obtained by the cross-validation of the (partial) likelihood (Goeman, 2010). If the estimated regression coefficient was not shrunk to zero, the variable was considered statistically significant.

We constructed the clinical index from the estimated penalized regression coefficients from the clinical model. Furthermore, the clinical-pharmacogenetic index was derived from the estimated penalized regression coefficients from the clinical-pharmacogenetic model. Indexes were defined as the linear predictors obtained from the penalized Cox model. Based on the penalized regression equations multivariate signatures for individual patients could be calculated. For both models the time-dependent ROC curves were constructed, where sensitivity, specificity, and tAUC were assessed. The larger the tAUC, the more predictive capacity the model had. When tAUC would be less or equal to 0.5, the model would have no predictive capacity. The predictive scoring system was estimated by selecting the threshold that provided the maximized sum of the cross-validated true positive rate and true negative rate. Cross-validation was applied on all the predictive accuracy estimates (AUC, true and false positive rates) to avoid biased and over-optimistic results. The apparent and cross-validated estimates were reported.

The predictive accuracy of the models was ranked as follows: AUC less than 0.6 was considered as worthless, between 0.6 and 0.7 as poor, between 0.7 and 0.8 as fair, between 0.8 and 0.9 as good and above 0.9 as excellent.

All of the statistical analyses were carried out using the R software (Goeman, 2010; Foucher and Danger, 2012; R Core Team, 2018).

## Results

### Patients’ Characteristics and Genotyping

Out of 231 PD patients included in the study, only 220 were included in the final analysis due to missing data. Among them 120 (54.5%) experienced MF, while 96 (43.6%) experienced dyskinesia. Median time from initiation of levodopa treatment to development of MF was 4.6 years (2.1–7.0), while median time from initiation of levodopa treatment to development of dyskinesia was 5.9 years (4.0–8.3). Median-follow up time for patients developing MF was 7.1 years (2.5–15.6), and median follow-up time for patients developing dyskinesia was 6.6 years (2.6–14.6). Patients’ characteristics are presented in the Table 1.

**Table 1:** Patients’ characteristics.

Characteristics	All patients (*N* = 220)	
Female sex	93 (42.3)	
Age at diagnosis (years)	62.2 (54.8–71.1)	
Time from diagnosis to initiation of levodopa treatment (years)	0 (0–1.4)	
Tremor-predominant PD	178 (80.9)	
Body side of disease initiation	Left	87 (39.5)
	Both	20 (9.1)
	Right	113 (51.4)
REM sleep behavior disorder	108 (49.1)	
Depression	97 (44.1)	
Constipation	97 (44.1)	
Olfactory dysfunction	96 (43.6)	
Beta-blockers	50 (22.7)	
Non-steroidal anti-inflammatory drugs	41 (18.6)	
Calcium channel blockers	37 (16.8)	
Statins	46 (20.9)	
Tobacco smoking (pack/year × years of smoking)	0 (0–8.4)	
Alcohol consumption (number of units in a lifetime)	447.2 (0–6955.0)	
Coffee consumption (cups per day)	1 (0–2)	
Motor fluctuations	120 (54.5)	
Dyskinesia	96 (43.6)	

*Categorical variables are presented as frequencies (percentages), whereas numerical variables are presented as median and interquartile range (IQR, first to third quartile).*

All of the patients were genotyped for 37 SNPs. Three of the SNPs (rs1060253, rs1060257, and rs1787467) deviated from HWE requirements (*p* < 0.05), which is why we excluded them from further analysis. In the case of *DDC* rs921451 and rs3837091 the frequencies also did not match the HWE requirements. However, frequencies for these two SNPs were not significantly different from frequencies reported in the 1000 Genomes EUR-CEU population (*p* = 0.705 and *p* = 0.097, respectively), which is the population that our patients are the most similar to in terms of ethnicity. These two SNPs were thus retained in the analysis. All other 32 SNPs were in HWE (*p* > 0.05).

### Univariate Cox Proportional Hazards Analysis for Prediction of Time to Occurrence of Motor Complications

Variables associated with time to occurrence of MF after initiation of levodopa treatment were: age at diagnosis (HR = 0.97; 95%CI = 0.96–0.99; *p* < 0.001), time from diagnosis to initiation of levodopa treatment (HR = 1.36; 95%CI = 1.25–1.49; *p* < 0.001), *NOS1* rs2293054 (GG: Ref.; AA: HR = 0.36; 95%CI = 0.13–1.00; *p* = 0.051), *DRD2* rs1799732 (CC: Ref.; –: HR = 8.89; 95%CI = 1.19–66.18; *p* = 0.033), and *DRD3* rs6280 (TT: Ref.; CC: HR = 2.04; 95%CI = 1.16–3.60; *p* = 0.014) (Supplementary Table 1).

Variables associated with time to occurrence of dyskinesia after initiation of levodopa treatment were: age at diagnosis (HR = 0.96; 95%CI = 0.95–0.98; *p* < 0.001), beta-blockers (HR = 0.60; 95%CI = 0.36–1.00; *p* = 0.051), time from diagnosis to initiation of levodopa treatment (HR = 1.23; 95%CI = 1.11–1.37, *p* < 0.001), *CAT* rs1001179 (GG: Ref.; AA: HR = 2.60; 95%CI = 1.17–5.79; *p* = 0.019), *SOD2* rs4880 (CC: Ref.; TT: HR = 0.54; 95%CI = 0.30–0.98; *p* = 0.043), *SLC22A1* rs628031 (GG: Ref.; GA: HR = 0.63; 95%CI = 0.40–1.00; *p* = 0.048; AA: HR = 0.53; 95%CI = 0.29–0.99; *p* = 0.047), *DRD2* rs1799732 (CC: Ref.; –: HR = 8.66; 95%CI = 1.16–64.86; *p* = 0.036), and *NRG1* rs3735781 (AA: Ref.; GA: HR = 0.65; 95%CI = 0.41–1.99; *p* = 0.051) (Supplementary Table 2).

### Clinical Multivariate Cox Analysis With LASSO Penalization for Prediction of Time to Occurrence of Motor Fluctuations

**FIGURE 1:**
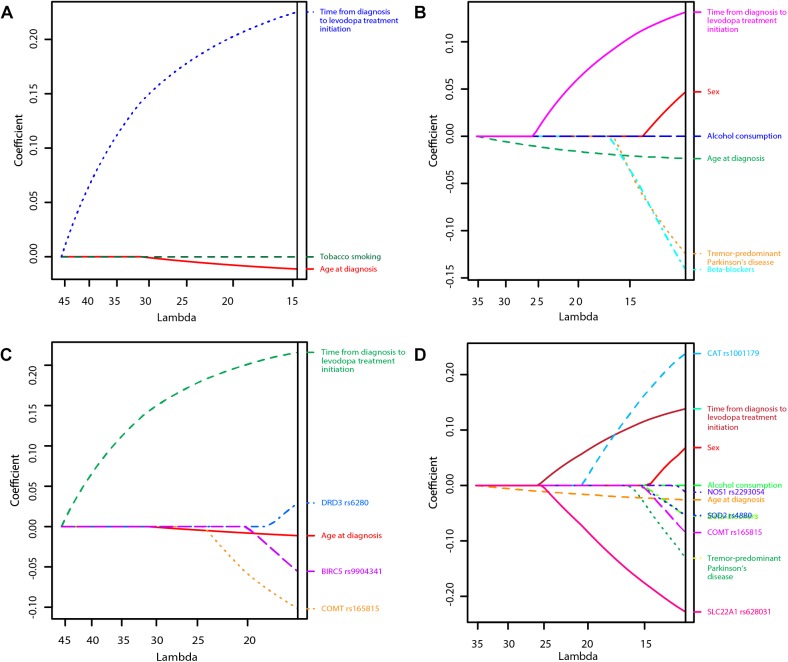
Least Absolute Shrinkage and Selection Operator (LASSO) penalized regression models of time to occurrence of motor fluctuations (MF) and dyskinesia after initiation of levodopa treatment (**A**: Clinical model for prediction of MF; **B**: Clinical model for prediction of dyskinesia; **C**: Clinical-pharmacogenetic model for prediction of MF; **D**: Clinical-pharmacogenetic model for prediction of dyskinesia). The highest predictive quality of the model was estimated at λ = 14.7, λ = 11.0, λ = 16.0, and λ = 12.2, respectively. Only significant variables are presented in the graphs as their regression coefficients were not shrunk to zero by λ.

The penalization method included the following variables in the predictive model: age at diagnosis (HR = 0.99), time from diagnosis to initiation of levodopa treatment (HR = 1.25), tobacco smoking (HR = 0.999974) (Figure 1A and Table 2).

**Table 2:** Variables selected by the LASSO penalized regression analysis for prediction of time to occurrence of motor fluctuations with the clinical model.

Selected variable	LASSO penalized regression^∗∗^	
	HR	Regression coefficient^∗^
Age at diagnosis	0.99	−0.011
Tobacco smoking	1.00^∗∗∗^	−2.59E-5
Time from diagnosis to initiation of levodopa treatment	1.25	0.225

*^∗^Regression coefficient is a natural logarithm of the HR. ^∗∗^Regression equation: multivariate signature for the patient = age at diagnosis ×-0.011 + tobacco smoking ×-2.59E-5 + time from diagnosis to initiation of levodopa treatment × 0.225. ^∗∗∗^The unrounded estimate of the coefficient is 0.999974. The direction of the effect can be identified from this unrounded number and from the negative sign of the corresponding regression coefficient.*

**FIGURE 2:**
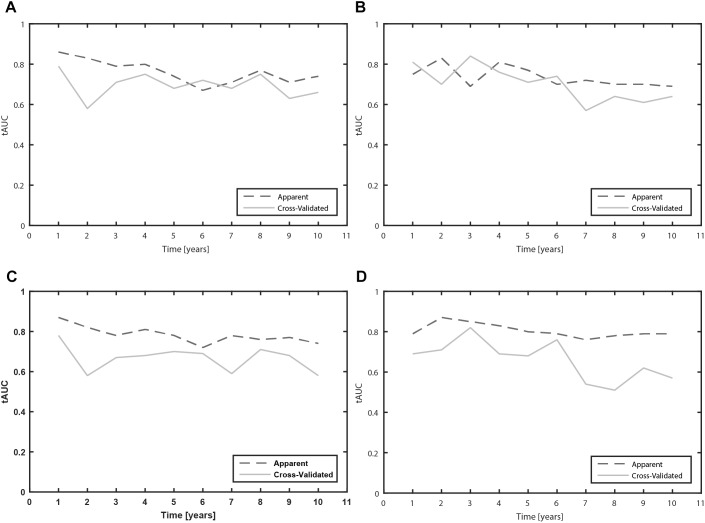
Time-dependent area under the receiver operating characteristic curves (tAUC) for prediction of MF and dyskinesia due to levodopa over the 10 year prognostic time (**A**: Clinical model for prediction of MF; **B**: Clinical model for prediction of dyskinesia; **C**: Clinical-pharmacogenetic model for prediction of MF; **D**: Clinical-pharmacogenetic model for prediction of dyskinesia).

Furthermore, we calculated the tAUC for each prognostic time from 1 to 10 years. We present the apparent and cross-validated tAUC. The cross-validated tAUC for prediction of time to occurrence of MF with clinical variables varied from 0.58 to 0.79 over the 10-year prognostic time, which means from worthless to fair. The results are presented in the Figure 2A.

**FIGURE 3:**
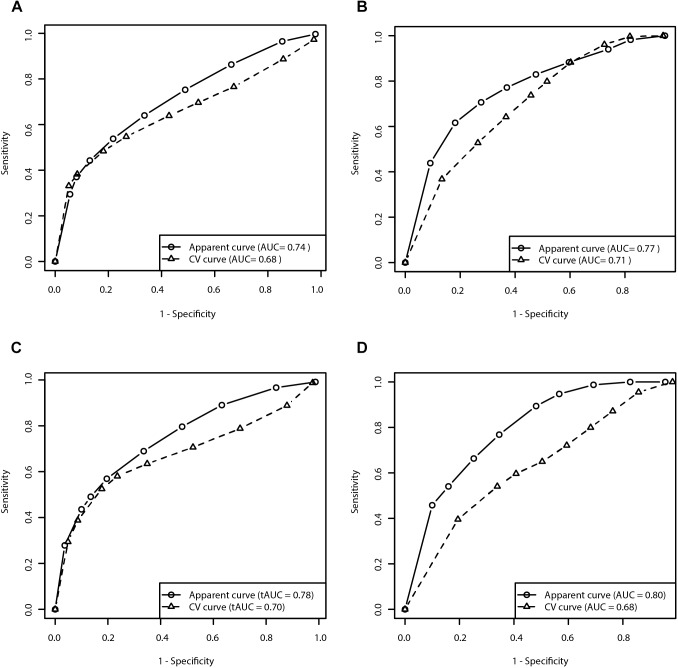
Receiver operating characteristic curves for prediction of the risk for development of MF and dyskinesia within the first 5 years of treatment (**A**: Clinical model for prediction of MF; **B**: Clinical model for prediction of dyskinesia; **C**: Clinical-pharmacogenetic model for prediction of MF; **D**: Clinical-pharmacogenetic model for prediction of dyskinesia). CV, cross-validated.

We evaluated the predictive capacity of the model in the first 5 years of treatment. ROC curve of the 5 year treatment period (apparent AUC = 0.74; cross-validated AUC = 0.68; sensitivity = 48.4%, specificity = 81.9%; Figure 3A) enabled us to construct the signature threshold of −0.26. Out of 146 patients with available data at 5 years of treatment 36 had the multivariate signature above the threshold, which means they were more likely to develop MF in the first 5 years of treatment. The model outcomes are presented in the Table 3.

**Table 3:** Outcomes of four constructed models for prediction of time to occurrence of motor complications after initiation of levodopa treatment.

	Motor fluctuations		Dyskinesia	
Patients with available data after 5 years of treatment with levodopa	146		135	
Patients with the adverse event after 5 years of treatment	69		33	

	**Clinical model**	**Clinical-pharmacogenetic model**	**Clinical model**	**Clinical-pharmacogenetic model**

Number of patients with increased risk for adverse event after 5 years predicted by the model	36	36	81	35
True positives	28	29	26	20
Number of false positives experiencing adverse event later in the follow-up time [median time to adverse event occurrence (years)]	6 (6.9)	6 (6.9)	46 (7.5)	14 (6.0)
False negatives	41	40	7	13

### Clinical Multivariate Cox Analysis With LASSO Penalization for Prediction of Time to Occurrence of Dyskinesia

LASSO penalization included the following clinical variables into the model: female sex (HR = 1.05), age at diagnosis (HR = 0.98), tremor-predominant PD (HR = 0.88), beta-blockers (HR = 0.87), alcohol consumption (HR = 0.999997), time from diagnosis to initiation of levodopa treatment (HR = 1.14) (Figure 1B and Table 4).

**Table 4:** Variables selected by the LASSO penalized regression analysis for prediction of time to occurrence of dyskinesia with the clinical model.

Selected variable	LASSO penalized regression^∗∗^	
	HR	Regression coefficient^∗^
Female sex	1.05	0.047
Age at diagnosis	0.98	−0.024
Tremor-predominant PD	0.88	−0.124
Beta-blockers	0.87	−0.141
Alcohol consumption	1.00^∗∗∗^	−3.44E–6
Time from diagnosis to initiation of levodopa treatment	1.14	0.131

*^∗^Regression coefficient is a natural logarithm of the HR. ^∗∗^Regression equation: multivariate signature for the patient = sex × 0.047 + age at diagnosis × −0.024 + tremor-predominant PD × −0.124 + beta-blockers × −0.141 + alcohol consumption × −3.44E-6 + time from diagnosis to initiation of levodopa treatment × 0.131. ^∗∗∗^The unrounded estimate of the coefficient is 0.999997. The direction of the effect can be identified from this unrounded number and from the negative sign of the corresponding regression coefficient.*

The cross-validated tAUC for prediction of dyskinesia with clinical variables varied from 0.61 to 0.84 over the 10-year prognostic time, which means from poor to good. The results are presented in the Figure 2B.

We evaluated the predictive capacity of the model in the first 5 years of treatment. ROC curve of the 5 year treatment period (apparent AUC = 0.77; cross-validated AUC = 0.71; sensitivity = 79.8%, specificity = 48.4%; Figure 3B) enabled us to construct the signature threshold of −1.51. Out of 135 patients 81 had a multivariate signature above the threshold, which means they were more likely to develop dyskinesia in the first 5 years of treatment. The model outcomes are presented in the Table 3.

### Clinical-Pharmacogenetic Multivariate Cox Analysis With LASSO Penalization for Prediction of Time to Occurrence of Motor Fluctuations

The penalization method included the following variables in the predictive model: age at diagnosis (HR = 0.99), time from diagnosis to initiation of levodopa treatment (HR = 1.24), *COMT* rs165815 (HR = 0.90), *DRD3* rs6280 (HR = 1.03), and *BIRC5* rs9904341 (HR = 0.95) (Figure 1C and Table 5). Among them only age at diagnosis, time from diagnosis to initiation of levodopa treatment, and *DRD3* rs6280 were previously identified as significant or nominally significant predictors by univariate analysis.

**Table 5:** Variables selected by the LASSO penalized regression analysis for prediction of time to occurrence of motor fluctuations compared to results of the univariate analysis.

Selected variable	Univariate analysis		LASSO penalized regression^∗∗^	
	HR	95%CI *p*-value	HR	Regression coefficient^∗^
Age at diagnosis	**0.97**	**0.96–0.99** **<0.001**	0.99	−0.011
Time from diagnosis to initiation of levodopa treatment	**1.36**	**1.25–1.49** **<0.001**	1.24	0.216
*COMT* rs165815	CC	0.52	0.21–1.32 0.168	0.90	−0.102
	CT	0.68	0.45–1.02 0.063		
	TT	Ref.		
*DRD3* rs6280	TT	Ref.	1.03	0.029
	TC	1.28	0.86–1.89 0.222		
	CC	**2.04**	**1.16–3.60** **0.014**		
*BIRC5* rs9904341	GG	Ref.	0.95	−0.056
	GC	0.77	0.52–1.16 0.212		
	CC	0.59	0.33–1.06 0.080		

*^∗^Regression coefficient is a natural logarithm of the HR. ^∗∗^Regression equation: multivariate signature for the patient = age at diagnosis ×-0.011 + time from diagnosis to initiation of levodopa treatment × 0.216 + COMT rs165815 ×-0.102 + DRD3 rs6280 × 0.029 + BIRC5 rs9904341 ×-0.056. Significant and nominally significant associations from the univariate analysis are written in bold text.*

**Table 6:** Variables selected by the LASSO penalized regression analysis for prediction of time to occurrence of dyskinesia compared to results of the univariate analysis.

Selected variable	Univariate analysis		LASSO penalized regression^∗∗^	
	HR	95%CI *p*-value	HR	Regression coefficient^∗^
Female sex	1.34	0.89–2.02 0.162	1.07	0.068
Age at diagnosis	**0.96**	**0.95–0.98** **<0.001**	0.97	−0.026
Tremor-predominant PD	0.69	0.44–1.08 0.105	0.88	−0.131
Beta-blockers	0.60	0.36–1.00 0.051	0.95	−0.056
Alcohol consumption	1.00	1.00–1.00 0.073	1.00^∗∗∗^	−1.27E-6
Time from diagnosis to initiation of levodopa treatment	**1.23**	**1.11–1.37** **<0.001**	1.15	0.138
*CAT* rs1001179	GG	Ref.	1.27	0.238
	GA	1.21	0.79–1.84 0.385		
	AA	**2.60**	**1.17–5.79** **0.019**		
*SOD2* rs4880	CC	Ref.	0.95	−0.054
	CT	0.69	0.43–1.12 0.132		
	TT	**0.54**	**0.30–0.98** **0.043**		
*NOS1* rs2293054	GG	Ref.	0.99	−0.012
	GA	0.83	0.54–1.27 0.390		
	AA	0.53	0.21–1.35 0.185		
*COMT* rs165815	CC	0.78	0.28–2.16 0.634	0.92	−0.085
	CT	0.73	0.47–1.15 0.171		
	TT	Ref.		
*SLC22A1* rs628031	GG	Ref.	0.80	−0.228
	GA	**0.63**	**0.40–1.00** **0.048**		
	AA	**0.53**	**0.29–0.99** **0.047**		

*^∗^Regression coefficient is a natural logarithm of the HR. ^∗∗^Regression equation: multivariate signature for the patient = sex × 0.068 + age at diagnosis × −0.026 + tremor-predominant PD × −0.131 + beta-blockers × 0.056 + alcohol consumption × −1.27E-6 + time from diagnosis to initiation of levodopa treatment × 0.138 + CAT rs1001179 × 0.238 + SOD2 rs4880 × −0.054 + NOS1 rs2293054 × −0.012 + COMT rs165815 × −0.085 + SLC22A1 rs628031 × −0.228. ^∗∗∗^The unrounded estimate of the coefficient is 0.999998. The direction of the effect can be identified from this unrounded number and from the negative sign of the corresponding regression coefficient. Significant and nominally significant associations from the univariate analysis are written in bold text.*

The cross-validated tAUC over the 10 year prognostic time varied from 0.58 to 0.78, which means that the predictive accuracy of the model varied from worthless to fair. The results are presented in the Figure 2C.

We have also checked the predictive capacity of the model after 5 years. With the ROC curve after 5 years of treatment (apparent AUC = 0.78, cross-validated AUC = 0.70; Figure 3C) we were able to construct a multivariate signature threshold of −0.33. The cross-validated sensitivity and specificity were 52.2 and 82.3%, respectively. Out of 146 patients with available data at 5 years of treatment, 36 patients had a multivariate signature above the selected threshold. These patients were considered more susceptible to MF development in the first 5 years of treatment. The model outcomes are presented in the Table 3.

### Clinical-Pharmacogenetic Multivariate Cox Analysis With LASSO Penalization for Prediction of Time to Occurrence of Dyskinesia

The penalization method included the following variables in the predictive model: sex (HR = 1.07), age at diagnosis (HR = 0.97), tremor-predominant PD (HR = 0.88), beta-blockers (HR = 0.95), alcohol consumption (HR = 0.999998), time from diagnosis to initiation of levodopa treatment (HR = 1.15), *CAT* rs1001179 (HR = 1.27), *SOD2* rs4880 (HR = 0.95), *NOS1* rs2293054 (HR = 0.99), *COMT* rs165815 (HR = 0.92), and *SLC22A1* rs628031 (HR = 0.80) (Figure 1D and Table 6). Among them only age at diagnosis, beta-blockers, time from diagnosis to initiation of levodopa treatment, *CAT* rs1001179, *SOD2* rs4880, and *SLC22A1* rs628031 were identified as significant or nominally significant predictors by univariate analysis.

The cross-validated tAUC for prediction of dyskinesia over the 10 year prognostic time varied from 0.51 to 0.82, which means that the predictive accuracy of the model varied from worthless to good. The results are presented in the Figure 2D.

We also checked the predictive capacity of the model after 5 years of levodopa treatment. With the ROC curve of the 5 year treatment (apparent AUC = 0.80, cross-validated AUC = 0.68; Figure 3D) we were able to construct a multivariate signature threshold of −1.33. The cross-validated sensitivity and specificity were 54.1 and 66.1%, respectively. Out of 135 patients with available data at 5 years of treatment, 35 patients had the multivariate signature above the selected threshold. These patients were considered more susceptible to dyskinesia development in the first 5 years of treatment. The model outcomes are presented in the Table 3.

## Discussion

This study presents a novel approach to predicting the occurrence of AEs of dopaminergic treatment in PD. It is also the first one to combine clinical and genetic explanatory variables for prediction of the time to occurrence of motor complications due to levodopa treatment. We built clinical and clinical-pharmacogenetic statistical models for prediction of motor complications based on 16 clinical variables and 34 genetic polymorphisms. Additionally, we compared predictive capacities of both types of models. No clinically important differences were found between the clinical and clinical-pharmacogenetic models, even though certain genetic factors were statistically significantly connected with time to occurrence of motor complications.

According to results of the univariate analysis, age at diagnosis was identified as inversely correlated with the time to occurrence of both MF and dyskinesia, which is in agreement with many previous reports. A younger age at onset is associated with early development of motor complications (You et al., 2018). Furthermore, longer time from diagnosis to initiation of levodopa treatment was associated with earlier development of motor complications. This may sound contradictory to the common belief about the preventive effect of the delayed onset of levodopa treatment. However, some recent studies have shown that a longer disease duration and higher levodopa daily dose play a major role in the development of levodopa related motor complications (Cilia et al., 2014; Eusebi et al., 2018). It is also believed that the storage and clearance of striatal dopamine are greatly reduced in the advanced PD. This means that brain concentrations of levodopa are based only on the drug-dosing cycle, which predisposes patients for motor complications (Calabresi et al., 2010; Yoo et al., 2018). More severe dopamine denervation, which increases with PD progression, also increases the risk for the occurrence of motor complications (You et al., 2018). Our observation that beta-blockers prolong time to dyskinesia development was not expected as beta-blockers increase the risk for PD development (Kalia and Lang, 2015). However, several experimental and clinical studies have observed an anti-dyskinetic effect of propranolol, a β-adrenergic receptor antagonist. Its anti-dyskinetic action could be mediated via attenuation of levodopa-induced extraphysiological efflux of dopamine (Bhide et al., 2015).

We observed three genetic factors that were associated with MF in the univariate analysis. *NOS1* genetic variability was already investigated in relation to dyskinesia development, but no association was found (Santos-Lobato et al., 2017). However, *NOS1* rs2293054 AA genotype was protective against early MF development in our cohort. Furthermore, *DRD2* rs1799732 – and *DRD3* rs6280 CC genotypes increased the chance for early MF development. *DRD2* rs1799732 has already been associated with the occurrence of dyskinesia (Politi et al., 2018). *DRD3* rs6280 has been associated with the earlier onset of dyskinesia as well as some other AEs (Politi et al., 2018; Redenšek et al., 2018). Several genetic factors were associated with the time to dyskinesia development in the univariate analysis. Associations of *CAT* rs1001179 and *SOD2* rs4880 with certain non-motor AEs of dopaminergic treatment were already detected in our previous study in the same cohort of patients, but their association with dyskinesia has never been reported before. Furthermore, *SLC22A1* rs628031 was already reported as protective against dyskinesia (Redenšek et al., 2019). We are the first to report an association of the neurodevelopmental *NRG1* rs3735781 GA genotype with the prolonged time to dyskinesia development, which corresponds to the NRG1’s protective role against neuroinflammation and oxidative stress (Xu et al., 2017).

Compared to univariate analysis, LASSO penalized regression models take into account all of the explanatory variables included in the analysis simultaneously. It may thus happen that different parameters appear as significantly associated with the phenotype compared to the univariate analysis.

The clinical-pharmacogenetic model for MF included age at diagnosis and time from diagnosis to initiation of levodopa treatment as clinical variables and *COMT* rs165815, *DRD3* rs6280, and *BIRC5* rs9904341 as genetic variables. *COMT* rs165815 has already been shown to protect from visual hallucinations (Redenšek et al., 2019), but has never been associated with MF before. *DRD3* rs6280 was consistently identified as a risk factor for MF in the univariate analysis and also in the model building process. *BIRC5* rs9904341 was correlated with increased expression at both mRNA and protein levels in cancer cells (Jenko et al., 2016). With its protective role against apoptosis and increased expression it might in fact protect from MF. Besides two clinical parameters that were already included in the clinical-pharmacogenetic model, tobacco smoking was also included in the clinical model. Its contribution seems to be rather small as its regression coefficient is small. Tobacco smoking appeared to be protective against MF. This was expected as previous reports indicate that tobacco smoking protects against PD (Kalia and Lang, 2015).

Several explanatory variables appeared to have an important effect on the time to dyskinesia occurrence. The clinical-pharmacogenetic model included sex, age at diagnosis, tremor-predominant PD, beta-blocker use, alcohol consumption, and time from diagnosis to initiation of levodopa treatment as clinical parameters. In concordance with previous reports, females had higher risk for early dyskinesia development (Eusebi et al., 2018). Tremor-predominant PD usually has milder course compared to akinetic-rigid form. According to our results and previous reports, the tremor-predominant form of PD also protects patients against early dyskinesia development (Eusebi et al., 2018). Based on our results alcohol consumption protects against early dyskinesia development and also protects against PD development according to previous reports (Kalia and Lang, 2015). Other clinical factors were already detected in the univariate analysis. Besides three genetic factors identified in the univariate analysis two other genetic factors were also included in the model, i.e., *NOS1* rs2293054 and *COMT* rs165815. *NOS1* rs2293054 influences splicing according to the SNP function prediction tool (Xu and Taylor, 2009), which might decrease protein production and thus protect against dyskinesia. *COMT* rs165815 has also never been associated with dyskinesia, which is similar to the case of MF. However, *COMT* rs4680 already showed association with this AE (Politi et al., 2018; Redenšek et al., 2018). Clinical model included the same clinical explanatory variables in the equation as the clinical-pharmacogenetic model.

We also evaluated the predictive capacity of the constructed models with the tAUC. The tAUC over the 10 year prognostic time was calculated, and the 5 year prognostic time was specifically analyzed. We focused on the 5 year time due to reports that approximately half of patients treated with levodopa experience motor complications after this period of treatment (Politi et al., 2018). We calculated the apparent and cross-validated AUC to avoid overoptimistic results due to overfitting. The cross-validated AUC for clinical model for MF was 0.68 and for clinical-pharmacogenetic model for MF 0.70. This shows no clinically important difference between the predictive capacities of the two models. Specificities (81.9 vs. 82.3%, respectively) and sensitivities (48.4 vs. 52.5%, respectively) did not differ tremendously either. Similarly, no clinically important difference in predictive ability was detected between clinical and clinical-pharmacogenetic models for dyskinesia as cross-validated AUC were 0.71 and 0.68, respectively. The predictive accuracies of the constructed models are poor to fair, which is far from enough for clinical use. One may conclude that the analyzed genetic factors do not significantly contribute to better prediction of the time to MF occurrence as predictive capacity did not improve when genetic factors were included in the analysis. However, specificity and sensitivity of the predictive models for dyskinesia differed rather a lot. Specificities for the clinical and clinical-pharmacogenetic models were 48.4 and 66.1%, respectively, while sensitivities were 79.8 and 54.1%, respectively. The specificity of the predictive model for dyskinesia improved when genetic factors were added to the analysis. This means that fewer patients were falsely identified to be at higher risk for dyskinesia. This indicates that genetic factors could play a role in predicting the time to occurrence of dyskinesia. However, further studies on subgroups of PD patients are warranted, stratified either on the basis of phenotype or on the basis of genetic variability. Certain genetic factors may only be important in one group of patients and not the other, which means that the effect is reduced or lost when the whole population is analyzed (Redenšek et al., 2017). Nevertheless, this is the first study that jointly evaluated the effect of different clinical and genetic factors on the time to motor complications development.

We calculated the thresholds for potentially clinically useful algorithm construction from the cross-validated ROC curves for each model after 5 years of treatment. From these thresholds we identified patients with increased risk for the early development of motor complications in the first 5 years of treatment. In majority of cases, identified patients were true positives meaning that they actually experienced the AE in the first 5 years of treatment (MF – clinical vs. clinical-pharmacogenetic model: 77.8 vs. 80.6%; dyskinesia – clinical vs. clinical-pharmacogenetic model: 32.1 vs. 57.1%). Some of them developed the AE later in the follow-up time, but very few patients identified to be at risk did not develop the AE during the follow-up time.

There are some limitations to the reported study. Although the sample size is comparable to sample sizes of other pharmacogenetic studies, this particular study would benefit from a larger sample as the number of events would increase in comparison to the number of explanatory variables (Day et al., 2018). Contributions of many factors are usually small and thus difficult to detect in a smaller population. However, this may also happen in larger studies due to phenotypic heterogeneity, which might increase with larger sample size. If the sample size was larger, we could perform an independent validation of the constructed models. Nevertheless, we performed cross-validation during the model construction process. Furthermore, if the sample size was larger, we would also be able to stratify patients according to their genetic characteristics or phenotype to identify parameters specifically important in subgroups of patients. To be able to divide patients into groups according to phenotype we would also need more detailed clinical data on the type of MF or dyskinesia. This was not feasible as this data was not consistently reported throughout medical records due to the retrospective nature of the study. A prospective study would have a better chance to detect more subtle effects of clinical and genetic parameters on the time to motor complications occurrence. A longer follow-up period would increase the quality of clinical data, especially the data on the outcomes in question. We did not include the levodopa dose in the analysis although it has an important effect on the occurrence of motor complications according to previous studies (Eusebi et al., 2018). We took into account only explanatory variables that are known at the initiation of levodopa therapy. Nevertheless, we also constructed models with the data on levodopa dose included (data not shown) and did not observe any improvement in predictive capacity compared to the models without this data.

However, there are a lot of strengths to this study that must not be overlooked. The studied population is genetically uniform (Mizzi et al., 2016). All the patients were recruited from the same department, which means they were treated according to the same guidelines. The reported study comprehensively assessed the simultaneous influence of several clinical and genetic parameters on the time to occurrence of motor complications after levodopa treatment initiation. The method of penalized regression used in this study permits the inclusion of correlated variables into the analysis. We also prevented overfitting and overoptimistic unrealistic results with cross-validation. Study was designed according to the pathway based approach as genetic parameters of five pathways already recognized as important in PD pathogenesis were included in the study (Redenšek et al., 2017).

## Conclusion

Here we report the associations of *NOS1* rs2293054, *DRD2* rs1799732, and *DRD3* rs6280 with time to occurrence of MF under the univariate analysis. Furthermore, *COMT* rs165815, *DRD3* rs6280, and *BIRC5* rs9904341 were associated with time to occurrence of MF within the clinical-pharmacogenetic predictive model. We report the associations of *DRD2* rs1799732, *NRG1* rs3735781, *CAT* rs1001179, *SOD2* rs4880, and *SLC22A1* rs628031 with time to occurrence of dyskinesia under the univariate analysis. The last three along with *COMT* rs165815, and *NOS1* rs2293054 were associated with time to occurrence of dyskinesia within the clinical-pharmacogenetic model. With this particular study we did not manage to show any important advantages of clinical-pharmacogenetic models over the clinical ones in the ability to predict the time to development of motor complications. Further analyses on larger independent samples are warranted to build predictive models on data from stratified groups of PD patients to decipher more subtle but important effects of genetic factors on the time to occurrence of motor complications.

## Data Availability

The datasets for this manuscript are not publicly available because the research is still ongoing. Requests to access the datasets should be directed to VD, vita.dolzan@mf.uni-lj.si.

## Ethics Statement

The study protocol was approved by the Slovenian Ethics Committee for Research in Medicine (KME 42/05/16). All subjects gave written informed consent in accordance with the Declaration of Helsinki.

## Author Contributions

SR, MT, and VD formed the study focus, and organized, and executed the study. SR performed the experiments. BJB carried out the statistical analysis. SR wrote the first draft of the manuscript. BJB, MT, and VD critically evaluated the manuscript. All authors have made a substantial intellectual contribution to this work and approved the final version of the manuscript for submission.

## Conflict of Interest Statement

The authors declare that the research was conducted in the absence of any commercial or financial relationships that could be construed as a potential conflict of interest.

## Abbreviations

AEs: adverse events
AUC: area under the receiver operating characteristic curve
CIs: confidence intervals
HRs: hazard ratios
HWE: Hardy–Weinberg equilibrium
LASSO: Least Absolute Shrinkage and Selection Operator
MF: motor fluctuations
PD: Parkinson’s disease
ROC: receiver operating characteristic
SNP: single nucleotide polymorphisms
tAUC: area under the time-dependent receiver operating characteristic curve.
