# Supplementary material for: Clinical-Pharmacogenetic Predictive Models for Time to Occurrence of Levodopa Related Motor Complications in Parkinson’s Disease
Source: Front Genet. 2019 May 16;10:461. doi: 10.3389/fgene.2019.00461 (PMC6532453; doi:10.3389/fgene.2019.00461)
Supplement: Supplementary file 1 [file Data_Sheet_1.docx]

**Supplementary table 1: Association of clinical and genetic variables with the time to occurrence of motor fluctuations after levodopa treatment initiation evaluated with univariate Cox regression and LASSO penalized Cox regression analyses**

| **Patient characteristic** | | | | | | | **Univariate analysis** | | **Penalized regression** | | |
| --- | --- | --- | --- | --- | --- | --- | --- | --- | --- | --- | --- |
|  |  |  |  |  |  |  | **HR** | **95%CI**  **p-value** | **Reg. coeff.** | **HR** | |
| Female sex | | | | | | | 0.19 | 0.82-1.72  0.364 | 0 | 1.00 | |
| Age at diagnosis (years) | | | | | | | **0.97** | **0.96-0.99**  **<0.001** | **-0.011** | **0.99** | |
| Time from diagnosis to levodopa treatment initiation (years) | | | | | | | **1.36** | **1.25-1.49**  **<0.001** | **0.216** | **1.24** | |
| Tremor-predominant PD | | | | | | | 0.92 | 0.60-1.41  0.706 | 0 | 1.00 | |
| Body side of disease initiation  (left = ref.) | | | Both | | | | 0.76 | 0.30-1.92  0.560 | 0 | 1.00 | |
|  |  |  | Right | | | | 1.15 | 0.79-1.67  0.478 |  |  |  |
| RBD | | | | | | | 1.12 | 0.78-1.62  0.531 | 0 | 1.00 | |
| Depression | | | | | | | 1.10 | 0.77-1.58  0.594 | 0 | 1.00 | |
| Constipation | | | | | | | 0.84 | 0.58-1.22  0.355 | 0 | 1.00 | |
| Olfactory dysfunction | | | | | | | 1.02 | 0.71-1.47  0.908 | 0 | 1.00 | |
| Beta-blockers | | | | | | | 0.65 | 0.41-1.03  0.066 | 0 | 1.00 | |
| NSAID | | | | | | | 0.86 | 0.53-1.40  0.554 | 0 | 1.00 | |
| Calcium channel blockers | | | | | | | 1.00 | 0.58-1.73  0.998 | 0 | 1.00 | |
| Statins | | | | | | | 0.68 | 0.39-1.21  0.190 | 0 | 1.00 | |
| Tobacco smoking (pack/year*years of smoking) | | | | | | | 1.00 | 1.00-1.00  0.117 | 0 | 1.00 | |
| Alcohol consumption (number of units in a lifetime) | | | | | | | 1.00 | 1.00-1.00  0.437 | 0 | 1.00 | |
| Coffee consumption (cups per day) | | | | | | | 0.89 | 0.72-1.09  0.263 | 0 | 1.00 | |
| **Gene** | **SNP** | **Genotype** | | **N** | **HWE** | **MAF*** |  | | | | |
| *NLRP3* | rs35829419 | CC | | 191 | 0.981 | 0.02 | Ref. | | 0 | | 1.00 |
|  |  | CA | | 28 |  |  | 1.02 | 0.57- 1.81  0.955 |  |  |  |
|  |  | AA | | 1 |  |  | 0.81 | 0.11- 5.82  0.832 |  |  |  |
| *CARD8* | rs2043211 | AA | | 106 | 0.172 | 0.32 | Ref. | | 0 | | 1.00 |
|  |  | AT | | 87 |  |  | 0.92 | 0.62 -1.38  0.711 |  |  |  |
|  |  | TT | | 27 |  |  | 1.31 | 0.77 -2.23  0.316 |  |  |  |
| *IL1B* | rs16944 | AA | | 23 | 0.532 | 0.49 | 0.96 | 0.53-1.74  0.899 | 0 | | 1.00 |
|  |  | AG | | 90 |  |  | 0.88 | 0.59 -1.29  0.502 |  |  |  |
|  |  | GG | | 107 |  |  | Ref. | |  |  |  |
|  | rs1143623 | GG | | 130 | 0.523 | 0.29 | Ref. | | 0 | | 1.00 |
|  |  | GC | | 76 |  |  | 0.83 | 0.57-1.23  0.355 |  |  |  |
|  |  | CC | | 14 |  |  | 1.20 | 0.57-2.50  0.629 |  |  |  |
| *TNFa* | rs1800629 | GG | | 152 | 0.291 | 0.09 | Ref. | | 0 | | 1.00 |
|  |  | GA | | 59 |  |  | 0.79 | 0.52-1.23  0.298 |  |  |  |
|  |  | AA | | 9 |  |  | 1.50 | 0.69-3.25  0.309 |  |  |  |
| *IL6* | rs1800795 | GG | | 62 | 0.123 | 0.14 | Ref. | | 0 | | 1.00 |
|  |  | GC | | 120 |  |  | 0.89 | 0.59-1.33  0.572 |  |  |  |
|  |  | CC | | 38 |  |  | 0.67 | 0.38-1.17  0.155 |  |  |  |
| *GPX1* | rs1050450 | CC | | 113 | 0.875 | 0.22 | Ref. | | 0 | | 1.00 |
|  |  | CT | | 90 |  |  | 1.27 | 0.87-1.86  0.217 |  |  |  |
|  |  | TT | | 17 |  |  | 0.82 | 0.40-1.68  0.586 |  |  |  |
| *CAT* | rs10836235 | CC | | 168 | 0.434 | 0.13 | Ref. | | 0 | | 1.00 |
|  |  | CT | | 47 |  |  | 0.87 | 0.57-1.35  0.546 |  |  |  |
|  |  | TT | | 5 |  |  | 0.38 | 0.09-1.54  0.174 |  |  |  |
|  | rs1001179 | GG | | 120 | 0.178 | 0.13 | Ref. | | 0 | | 1.00 |
|  |  | GA | | 90 |  |  | 0.95 | 0.65-1.38  0.778 |  |  |  |
|  |  | AA | | 10 |  |  | 1.00 | 0.43-2.32  1.000 |  |  |  |
| *SOD2* | rs4880 | CC | | 62 | 0.610 | 0.41 | Ref. | | 0 | | 1.00 |
|  |  | CT | | 106 |  |  | 0.70 | 0.45-1.08  0.106 |  |  |  |
|  |  | TT | | 52 |  |  | 0.63 | 0.37-1.08  0.096 |  |  |  |
| *NOS1* | rs2293054 | GG | | 118 | 0.951 |  | Ref. | | 0 | | 1.00 |
|  |  | GA | | 86 |  | 0.25 | 1.33 | 0.92-1.94  0.129 |  |  |  |
|  |  | AA | | 16 |  |  | **0.36** | **0.13-1.00**  **0.051** |  |  |  |
|  | rs2682826 | GG | | 107 | 0.096 | 0.26 | Ref. | | 0 | | 1.00 |
|  |  | GA | | 100 |  |  | 1.06 | 0.73-1.54  0.758 |  |  |  |
|  |  | AA | | 13 |  |  | 0.57 | 0.26-1.27  0.171 |  |  |  |
| *COMT* | rs4680 | GG | | 58 | 0.179 | 0.37 | Ref. | | 0 | | 1.00 |
|  |  | GA | | 100 |  |  | 1.26 | 0.80-2.00  0.318 |  |  |  |
|  |  | AA | | 62 |  |  | 1.30 | 0.80-2.11  0.283 |  |  |  |
|  | rs165815 | CC | | 8 | 0.994 | 0.38 | 0.52 | 0.21-1.32  0.168 | **-0.102** | | **0.90** |
|  |  | CT | | 68 |  |  | 0.68 | 0.45-1.02  0.063 |  |  |  |
|  |  | TT | | 144 |  |  | Ref. | |  |  |  |
| *DDC* | rs921451 | TT | | 96 | 0.004 | 0.35 | Ref. | | 0 | | 1.00 |
|  |  | CT | | 83 |  |  | 0.91 | 0.61-1.35  0.624 |  |  |  |
|  |  | CC | | 41 |  |  | 0.92 | 0.55-1.54  0.762 |  |  |  |
|  | rs3837091 | AGAGAGAG | | 116 | 0.008 | 0.29 | Ref. | | 0 | | 1.00 |
|  |  | AGAG- | | 76 |  |  | 1.10 | 0.75-1.62  0.623 |  |  |  |
|  |  | -- | | 28 |  |  | 0.75 | 0.40-1.40  0.363 |  |  |  |
| *MAOB* | rs1799836 | A, AA | | 101 | 0.684 | 0.46 | Ref. | | 0 | | 1.00 |
|  |  | AG | | 50 |  |  | 1.20 | 0.76-1.91  0.433 |  |  |  |
|  |  | G, GG | | 69 |  |  | 0.82 | 0.53-1.26  0.360 |  |  |  |
| *SLC6A3* | rs6347 | AA | | 117 | 0.347 | 0.30 | Ref. | | 0 | | 1.00 |
|  |  | AG | | 83 |  |  | 1.07 | 0.73-1.58  0.723 |  |  |  |
|  |  | GG | | 20 |  |  | 1.26 | 0.66-2.41  0.478 |  |  |  |
|  | rs1042098 | TT | | 118 | 0.409 | 0.30 | Ref. | | 0 | | 1.00 |
|  |  | TC | | 83 |  |  | 1.20 | 0.82-1.75  0.353 |  |  |  |
|  |  | CC | | 19 |  |  | 0.87 | 0.45-1.71  0.695 |  |  |  |
|  | rs393795 | GG | | 139 | 0.380 | 0.37 | Ref. | | 0 | | 1.00 |
|  |  | GT | | 69 |  |  | 0.85 | 0.57-1.27  0.436 |  |  |  |
|  |  | TT | | 12 |  |  | 0.88 | 0.38-2.02  0.765 |  |  |  |
| *SLC22A1* | rs628031 | GG | | 92 | 0.633 | 0.31 | Ref. | | 0 | | 1.00 |
|  |  | GA | | 98 |  |  | 0.79 | 0.53-1.19  0.265 |  |  |  |
|  |  | AA | | 30 |  |  | 0.92 | 0.56-1.52  0.749 |  |  |  |
| *SLC18A2* | rs14240 | TT | | 57 | 0.260 | 0.45 | Ref. | | 0 | | 1.00 |
|  |  | TC | | 118 |  |  | 1.23 | 0.80-1.88  0.347 | 0 | | 1.00 |
|  |  | CC | | 45 |  |  | 0.84 | 0.48-1.45  0.523 |  |  |  |
| *SV2C* | rs1423099 | CC | | 20 | 0.518 | 0.44 | 0.78 | 0.39-1.58  0.498 | 0 | | 1.00 |
|  |  | CT | | 86 |  |  | 1.13 | 0.78-1.65  0.522 |  |  |  |
|  |  | TT | | 114 |  |  | Ref. | |  |  |  |
| *DRD2* | rs1801028 | CC | | 210 | 0.730 | 0.03 | Ref. | | 0 | | 1.00 |
|  |  | GC | | 10 |  |  | 1.00 | 0.41-2.46  0.999 |  |  |  |
|  |  | GG | | 0 |  |  | / | / |  |  |  |
|  | rs1799732 | CC | | 181 | 0.943 | 0.24 | Ref. | | 0 | | 1.00 |
|  |  | C- | | 37 |  |  | 0.88 | 0.52-1.47  0.619 |  |  |  |
|  |  | -- | | 2 |  |  | **8.89** | **1.19-66.18**  **0.033** |  |  |  |
| *DRD3* | rs6280 | TT | | 105 | 0.096 | 0.49 | Ref. | | **0.029** | | **1.03** |
|  |  | TC | | 86 |  |  | 1.28 | 0.86-1.89  0.222 |  |  |  |
|  |  | CC | | 29 |  |  | **2.04** | **1.16-3.60**  **0.014** |  |  |  |
| *BDNF* | rs6265 | GG | | 132 | 0.051 | 0.20 | Ref. | | 0 | | 1.00 |
|  |  | GA | | 83 |  |  | 1.20 | 0.83-1.75  0.337 |  |  |  |
|  |  | AA | | 5 |  |  | 1.44 | 0.35-5.91  0.614 |  |  |  |
| *NOTCH4* | rs367398 | GG | | 91 | 0.847 | 0.43 | Ref. | | 0 | | 1.00 |
|  |  | GA | | 102 |  |  | 0.80 | 0.54-1.17  0.242 |  |  |  |
|  |  | AA | | 27 |  |  | 0.87 | 0.47-1.59  0.638 |  |  |  |
| *NRG1* | rs10503929 | TT | | 135 | 0.416 | 0.07 | Ref. | | 0 | | 1.00 |
|  |  | TC | | 72 |  |  | 0.83 | 0.56-1.22  0.343 |  |  |  |
|  |  | CC | | 13 |  |  | 0.86 | 0.39-1.87  0.701 |  |  |  |
| *NRG1* | rs3735782 | AA | | 41 | 0.398 | 0.42 | 1.28 | 0.76-2.16  0.344 | 0 | | 1.00 |
|  |  | AC | | 115 |  |  | 1.04 | 0.69-1.57  0.858 |  |  |  |
|  |  | CC | | 64 |  |  | Ref. | |  |  |  |
| *NRG1* | rs3735781 | AA | | 88 | 0.614 | 0.32 | Ref. | | 0 | | 1.00 |
|  |  | GA | | 105 |  |  | 1.21 | 0.82-1.79  0.348 |  |  |  |
|  |  | GG | | 27 |  |  | 1.33 | 0.76-2.33  0.324 |  |  |  |
| *NRG1* | rs3924999 | CC | | 109 | 0.544 | 0.39 | Ref. | | 0 | | 1.00 |
|  |  | TC | | 89 |  |  | 0.83 | 0.57-1.22  0.347 |  |  |  |
|  |  | TT | | 22 |  |  | 0.87 | 0.48-1.59  0.658 |  |  |  |
| *BIRC5* | rs9904341 | GG | | 82 | 0.780 | 0.39 | Ref. | | **-0.056** | | **0.95** |
|  |  | GC | | 103 |  |  | 0.77 | 0.52-1.16  0.212 |  |  |  |
|  |  | CC | | 35 |  |  | 0.59 | 0.33-1.06  0.080 |  |  |  |
| *BIRC5* | rs8073069 | GG | | 141 | 0.798 | 0.34 | Ref. | | 0 | | 1.00 |
|  |  | CG | | 71 |  |  | 0.98 | 0.67-1.45  0.929 |  |  |  |
|  |  | CC | | 8 |  |  | 1.00 | 0.31-3.19  0.997 |  |  |  |

* According to dbSNP (Sherry et al., 2001)

**Supplementary table 2: Association of clinical and genetic variables with the time to occurrence of dyskinesia after levodopa treatment initiation evaluated with univariate Cox regression and LASSO penalized Cox regression analyses**

| **Patient characteristic** | | | | | | **Univariate analysis** | | **Penalized regression** | |
| --- | --- | --- | --- | --- | --- | --- | --- | --- | --- |
|  |  |  |  |  |  | **HR** | **95%CI**  **p-value** | **Regression coefficient** | **HR** |
| Female sex | | | | | | 1.34 | 0.89-2.02  0.162 | **0.068** | **1.07** |
| Age at diagnosis (years) | | | | | | **0.96** | **0.95-0.98**  **3.99e-05** | **-0.026** | **0.97** |
| Time from diagnosis to levodopa treatment initiation (years) | | | | | | **1.23** | **1.11-1.37**  **5.87e-05** | **0.138** | **1.15** |
| Tremor-predominant PD | | | | | | 0.69 | 0.44-1.08  0.105 | **-0.131** | **0.88** |
| Body side of disease initiation  (left = ref.) | | | Both | | | 1.04 | 0.41-2.66  0.937 | 0 | 1.00 |
|  |  |  | Right | | | 1.07 | 0.70-1.64  0.746 |  |  |
| RBD | | | | | | 0.97 | 0.65-1.46  0.902 | 0 | 1.00 |
| Depression | | | | | | 1.27 | 0.85-1.89  0.251 | 0 | 1.00 |
| Constipation | | | | | | 0.95 | 0.64-1.43  0.818 | 0 | 1.00 |
| Olfactory dysfunction | | | | | | 0.92 | 0.6-1.37  0.679 | 0 | 1.00 |
| Beta-blockers | | | | | | **0.60** | **0.36-1.00**  **0.051** | **-0.056** | **0.95** |
| NSAID | | | | | | 0.79 | 0.45-1.37  0.401 | 0 | 1.00 |
| Calcium channel blockers | | | | | | 0.60 | 0.29-1.25  0.174 | 0 | 1.00 |
| Statins | | | | | | 0.72 | 0.39-1.33  0.299 | 0 | 1.00 |
| Tobacco smoking (pack/year*years of smoking) | | | | | | 1.00 | 1.00-1.00  0.591 | 0 | 1.00 |
| Alcohol consumption (number of units in a lifetime) | | | | | | 1.00 | 1.00-1.00  0.073 | **-0.127e-6** | **1.00*** |
| Coffee consumption (cups per day) | | | | | | 1.11 | 0.90-1.38  0.318 | 0 | 1.00 |
|  | | **Gene** | | **SNP** |  |  | | | |
|  |  | *NLRP3* | | rs35829419 | CC | Ref. | | 0 | 1.00 |
|  |  |  |  |  | CA | 1.22 | 0.66-2.25  0.521 |  |  |
|  |  |  |  |  | AA | 0.98 | 0.14-7.07  0.983 |  |  |
|  |  | *CARD8* | | rs2043211 | AA | Ref. | | 0 | 1.00 |
|  |  |  |  |  | AT | 1.22 | 0.79-1.90  0.373 |  |  |
|  |  |  |  |  | TT | 1.47 | 0.81-2.67  0.203 |  |  |
|  |  | *IL1B* | | rs16944 | AA | 1.00 | 0.5- 1.91  0.994 | 0 | 1.00 |
|  |  |  |  |  | AG | 0.96 | 0.62-1.47  0.845 |  |  |
|  |  |  |  |  | GG | Ref. | |  |  |
|  |  |  |  | rs1143623 | GG | Ref. | | 0 | 1.00 |
|  |  |  |  |  | GC | 1.01 | 0.66-1.55  0.962 |  |  |
|  |  |  |  |  | CC | 1.17 | 0.53-2.58  0.706 |  |  |
|  |  | *TNFa* | | rs1800629 | GG | Ref. | | 0 | 1.00 |
|  |  |  |  |  | GA | 1.02 | 0.64-1.62  0.940 |  |  |
|  |  |  |  |  | AA | 2.33 | 0.92-5.88  0.073 |  |  |
|  |  | *IL6* | | rs1800795 | GG | Ref. | | 0 | 1.00 |
|  |  |  |  |  | GC | 0.96 | 0.61-1.51  0.854 |  |  |
|  |  |  |  |  | CC | 1.03 | 0.55-1.91  0.937 |  |  |
|  |  | *GPX1* | | rs1050450 | CC | Ref. | | 0 | 1.00 |
|  |  |  |  |  | CT | 1.32 | 0.87-2.02  0.191 |  |  |
|  |  |  |  |  | TT | 0.82 | 0.37-1.83  0.633 |  |  |
|  | *CAT* | | rs10836235 | CC | Ref. | | 0 | 1.00 |  |
|  |  |  |  | CT | 1.09 | 0.69-1.74  0.705 |  |  |  |
|  |  |  |  | TT | 0.72 | 0.18-2.96  0.654 |  |  |  |
|  |  |  | rs1001179 | GG | Ref. | | **0.238** | **1.27** |  |
|  |  |  |  | GA | 1.21 | 0.79-1.84  0.385 |  |  |  |
|  |  |  |  | AA | **2.60** | **1.17-5.79**  **0.019** |  |  |  |
| *SOD2* | | rs4880 | CC | Ref. | | **-0.054** | **0.95** |  |  |
|  |  |  | CT | 0.69 | 0.43-1.12  0.132 |  |  |  |  |
|  |  |  | TT | **0.54** | **0.30-0.98**  **0.043** |  |  |  |  |
| *NOS1* | | rs2293054 | GG | Ref. | | **-0.012** | **0.99** |  |  |
|  |  |  | GA | 0.83 | 0.54-1.27  0.390 |  |  |  |  |
|  |  |  | AA | 0.53 | 0.21-1.35  0.185 |  |  |  |  |
|  |  | rs2682826 | GG | Ref. | | 0 | 1.00 |  |  |
|  |  |  | GA | 1.00 | 0.66-1.52  0.996 |  |  |  |  |
|  |  |  | AA | 0.55 | 0.22-1.39  0.203 |  |  |  |  |
| *COMT* | | rs4680 | GG | Ref. | | 0 | 1.00 |  |  |
|  |  |  | GA | 0.73 | 0.44-1.20  0.211 |  |  |  |  |
|  |  |  | AA | 0.79 | 0.47-1.31  0.358 |  |  |  |  |
|  |  | rs165815 | CC | 0.78 | 0.28-2.16  0.634 | **-0.085** | **0.92** |  |  |
|  |  |  | CT | 0.73 | 0.47-1.15  0.171 |  |  |  |  |
|  |  |  | TT | Ref. | |  |  |  |  |
| *DDC* | | rs921451 | TT | Ref. | | 0 | 1.00 |  |  |
|  |  |  | CT | 0.75 | 0.48-1.17  0.210 |  |  |  |  |
|  |  |  | CC | 0.95 | 0.55-1.66  0.865 |  |  |  |  |
|  |  | rs3837091 | AGAGAGAG | Ref. | | 0 | 1.00 |  |  |
|  |  |  | AGAG- | 0.99 | 0.65-1.51  0.949 |  |  |  |  |
|  |  |  | -- | 0.73 | 0.35-1.49  0.380 |  |  |  |  |
| *MAOB* | | rs1799836 | A, AA | Ref. | | 0 | 1.00 |  |  |
|  |  |  | AG | 1.16 | 0.70-1.95  0.563 |  |  |  |  |
|  |  |  | G, GG | 0.84 | 0.519-1.35  0.466 |  |  |  |  |
| *SLC6A3* | | rs6347 | AA | Ref. | | 0 | 1.00 |  |  |
|  |  |  | AG | 0.98 | 0.64-1.50  0.915 |  |  |  |  |
|  |  |  | GG | 0.92 | 0.44-1.94  0.827 |  |  |  |  |
|  |  | rs1042098 | TT | Ref. | | 0 | 1.00 |  |  |
|  |  |  | TC | 1.17 | 0.77-1.77  0.460 |  |  |  |  |
|  |  |  | CC | 0.43 | 0.17-1.08  0.072 |  |  |  |  |
|  |  | rs393795 | GG | Ref. | | 0 | 1.00 |  |  |
|  |  |  | GT | 1.11 | 0.72-1.73  0.641 |  |  |  |  |
|  |  |  | TT | 1.58 | 0.680-3.67  0.288 |  |  |  |  |
| *SLC22A1* | | rs628031 | GG | Ref. | | **-0.228** | **0.80** |  |  |
|  |  |  | GA | **0.63** | **0.40-1.00**  **0.048** |  |  |  |  |
|  |  |  | AA | **0.53** | **0.29-0.99**  **0.047** |  |  |  |  |
| *SLC18A2* | | rs14240 | TT | Ref. | | 0 | 1.00 |  |  |
|  |  |  | TC | 1.04 | 0.65-1.66  0.859 |  |  |  |  |
|  |  |  | CC | 0.87 | 0.47-1.60  0.657 |  |  |  |  |
| *SV2C* | | rs1423099 | CC | 1.14 | 0.56-2.34  0.719 | 0 | 1.00 |  |  |
|  |  |  | CT | 1.14 | 0.75-1.74  0.549 |  |  |  |  |
|  |  |  | TT | Ref. | |  |  |  |  |
| *DRD2* | | rs1801028 | CC | Ref. | | 0 | 1.00 |  |  |
|  |  |  | GC | 0.77 | 0.24-2.44  0.659 |  |  |  |  |
|  |  |  | GG | / | / |  |  |  |  |
|  |  | rs1799732 | CC | Ref. | | 0 | 1.00 |  |  |
|  |  |  | C- | 0.75 | 0.40-1.42  0.380 |  |  |  |  |
|  |  |  | -- | **8.66** | **1.16-64.86**  **0.036** |  |  |  |  |
| *DRD3* | | rs6280 | TT | Ref. | | 0 | 1.00 |  |  |
|  |  |  | TC | 1.00 | 0.65-1.53  0.993 |  |  |  |  |
|  |  |  | CC | 1.68 | 0.84-3.36  0.145 |  |  |  |  |
| *BDNF* | | rs6265 | GG | Ref. | | 0 | 1.00 |  |  |
|  |  |  | GA | 1.09 | 0.71-1.66  0.686 |  |  |  |  |
|  |  |  | AA | 1.09 | 0.15-8.01  0.930 |  |  |  |  |
| *NOTCH4* | | rs367398 | GG | Ref. | | 0 | 1.00 |  |  |
|  |  |  | GA | 1.02 | 0.66-1.55  0.947 |  |  |  |  |
|  |  |  | AA | 0.81 | 0.40-1.63  0.555 |  |  |  |  |
| *NRG1* | | rs10503929 | TT | Ref. | | 0 | 1.00 |  |  |
|  |  |  | TC | 1.03 | 0.67-1.58  0.889 |  |  |  |  |
|  |  |  | CC | 1.12 | 0.51-2.45  0.787 |  |  |  |  |
| *NRG1* | | rs3735782 | AA | 0.88 | 0.47-1.16  0.637 | 0 | 1.00 |  |  |
|  |  |  | AC | 0.74 | 0.51-1.51  0.192 |  |  |  |  |
|  |  |  | CC | Ref. | |  |  |  |  |
| *NRG1* | | rs3735781 | AA | Ref. | | 0 | 1.00 |  |  |
|  |  |  | GA | **0.65** | **0.41-1.00**  **0.051** |  |  |  |  |
|  |  |  | GG | 0.90 | 0.50-1.61  0.712 |  |  |  |  |
| *NRG1* | | rs3924999 | CC | Ref. | | 0 | 1.00 |  |  |
|  |  |  | TC | 1.27 | 0.82-1.95  0.280 |  |  |  |  |
|  |  |  | TT | 0.88 | 0.45-1.75  0.725 |  |  |  |  |
| *BIRC5* | | rs9904341 | GG | Ref. | | 0 | 1.00 |  |  |
|  |  |  | GC | 0.81 | 0.52-1.27  0.361 |  |  |  |  |
|  |  |  | CC | 0.90 | 0.49-1.66  0.745 |  |  |  |  |
| *BIRC5* | | rs8073069 | GG | Ref. | | 0 | 1.00 |  |  |
|  |  |  | CG | 0.78 | 0.50-1.22  0.276 |  |  |  |  |
|  |  |  | CC | 1.22 | 0.38-3.91  0.736 |  |  |  |  |

*The unrounded estimate of the coefficient is 0.999998. The direction of the effect can be identified from this unrounded number and from the negative sign of the corresponding regression coefficient.
